# Lessons from Neurotrauma Treatment Simulation Center (NTSC) – The neurosurgeon's perspective

**DOI:** 10.25122/jml-2022-1017

**Published:** 2022-07

**Authors:** Vlad Adrian Padurean, Dafin Fior Muresanu

**Affiliations:** 1. Neurosurgery Department, County Emergency Hospital, Cluj-Napoca, Romania; 2. RoNeuro Institute for Neurological Research and Diagnostic, Cluj-Napoca, Romania; 3. Department of Neuroscience, Iuliu Hatieganu University of Medicine and Pharmacy, Cluj-Napoca, Romania

The Neurotrauma Treatment Simulation Center (NTSC) Program, which took place on 16^th^-20^th^ May 2022 in Vienna, Austria, brought together delegates from 6 countries, including Romania, Slovakia, Egypt, Mexico, the Philippines, and Poland. The NTSC represented a training program developed and organized by faculty members from leading healthcare institutions (Allgemeines Krankenhaus Wien (AKH), Landesklinikum Wiener Neustadt, Klinik Pirawarth, Klinik Floridsdorf).

For most participants, it was the first scientific event not exclusively focused on a singular speciality, but it brought together specialists from a diverse range of neurotrauma-related fields like neurosurgery, traumatology, neurology, anesthesiology, neurorehabilitation, emergency medicine.

Considering this, the NTSC represented a unique experience both for witnessing the closely related medical specialties at work, and for bringing one's contribution to the management of neurotrauma. Surgical specialties, in particular, are considered quite secluded from medical ones, probably because surgeons are the only ones knowing what is being performed in the narrow operating field during surgery, so the impact of this type of event, especially of multidisciplinary training or simulations, such as the one at Floridsdorf Klinik, is further capitalized in the current and future practice of neurosurgeons.

A common practice in treating a neurosurgical case, from the emergency unit to the operating room and the rehabilitation center, is that the specialists involved have their own narrow tasks and focus. Inherent borders in communication and collaboration of specialists involved in the patient's pathway are an intrinsic issue. In most cases, this approach is appropriate for the patient, yet the NTSC program revealed multidisciplinarity to be of most advantage to the entire management of the patient, especially when dealing with complex trauma.

The practice of “peeking over the fence” to the closely related medical specialties and bringing one's contribution is needed for inpatient care and outpatient rehabilitation. Although plenty of cases can be managed in a segmented approach, in critical situations, neurosurgeons, anesthesiologists and neurologists are overwhelmed by the complexity of the case, and borderless communication becomes essential.

The organizers at NTSC tried to precisely bridge the communication gap across medical specialties by successfully implementing the principle of a heterogenous working group, including neurosurgeons, anesthesiologists, and neurologists. In a simulated practice, they had to experience the entire rescue chain in a country like Austria, starting with the first-line emergency response in the prehospital phase at Christophorus 3 Airbase in Wiener am Neustadt to the treatment within AKH Wien, and all the way towards the later phases of neurological care and rehabilitation at Klinik Floridsdorf and Bad Pirawarth Center. In other words, managing a multifaceted situation is precisely dealing with the unknown and figuring out the way through it by having just a few guiding instruments or principles at hand along the way.

Such trainings could find their applicability in low- and middle-income countries by eliminating both physical and communication borders that are still present, *i.e*., bringing specialists with various backgrounds more frequently together to share the best approach and practices. The experience at AKH highlighted the need for improved teamwork and collaboration among neurosurgery, neurology, anesthesiology (and other specialities); hopefully, also in East-European countries like Romania, prerequisites would be set in place for the development of other specializations, such as trauma surgery.

Furthermore, alongside technical advancements in the hospital and clinics from NTSC, the human factor, often overlooked, is a requisite to the entire rescue chain for patients with neurotrauma. In other words, the accent must be placed on educating people to work with others, especially to learn to better deal with high-pressure situations. Although equipping hospitals, especially in low-middle-income countries, is of utmost importance, developing networks in the neurotrauma chain can lead to an even higher standard of care.

To conclude, this type of hands-on training and communication within a multidisciplinary working group goes beyond the theoretical approach frequently found at the university level. Moreover, this multimodal approach cannot be assimilated and capitalized without a strong desire for personal growth and continuous development of know-how.

